# Advances and clinical potential of epigenome editing

**DOI:** 10.1007/s00018-026-06311-0

**Published:** 2026-06-27

**Authors:** Insung Choi, Sueon Kim, Inwha Baek

**Affiliations:** 1 Department of Regulatory Science, Graduate School, Kyung Hee University, Seoul, 02447 Korea; 2 Institute of Regulatory Innovation through Science (IRIS), Kyung Hee University, Seoul, 02447 Korea; 3 College of Pharmacy, Kyung Hee University, Seoul, 02447 Korea

**Keywords:** Epigenome editing, Precision medicine, Transcriptional control, Chromatin landscape, Translational medicine

## Abstract

Epigenome editing has emerged as a powerful platform to modulate gene expression in a precise and reversible manner. Recent advances have significantly improved the efficiency, specificity, and durability of epigenome editing systems, enabling fine-tuned transcriptional control. Building on these developments, epigenome editing platforms are now being explored for therapeutic applications. In this review, we summarize the evolution of clustered regularly interspaced short palindromic repeats (CRISPR)-based epigenome editing technologies, highlighting key improvements in effector modules. We then discuss the disease models in which epigenome editing has been applied, including monogenic disorders, cancer, neurological diseases, and chronic diseases. These examples demonstrate the broad therapeutic promise of targeted epigenetic modulation across diverse pathological contexts. Finally, we tackle key barriers to clinical translation, including cell-type and chromatin context-specific design, in vivo delivery, and multi-gene targeting for complex disease. Collectively, this review underscores the potential of epigenome editing as a versatile platform for precision medicine.

## Background

Epigenetic dysregulation is closely associated with the development and progression of numerous human diseases [1, 2]. In Alzheimer’s disease, key neuronal genes are aberrantly repressed via epigenetic alterations, including CpG island methylation and histone H3 trimethylation at lysine 27 (H3K27me3) and at lysine 9 (H3K9me3) [3]. In cancer, epigenetic silencing of tumor suppressor genes and/or overexpression of oncogenes has been widely reported [4–6]. In some disorders, epigenetic aberrations in a single gene serve as the primary cause of disease. For example, Fragile X syndrome results from abnormal expansion of CGG repeats in the 5′ untranslated region of *FMR1*, which leads to promoter hypermethylation and transcriptional silencing [7].

Small-molecule-based epigenetic drugs, commonly referred to as epidrugs, have been used clinically to treat various cancers. They inhibit endogenous epigenetic effectors, including DNA methyltransferases (DNMTs), histone deacetylases (HDACs), and bromodomain and extra-terminal domains, but lack target and cell-type specificity, resulting in off-target effects and high toxicity [8]. Epigenome editing has emerged as a promising therapeutic modality that can overcome limitations of epidrugs. By modulating the epigenetic landscape, including DNA methylation, histone modifications, and chromatin accessibility in a locus-specific, reversible, inducible, and durable manner, epigenome editing offers a safer and more targeted alternative [9, 10]. Unlike genome editing, it reprograms transcription without DNA sequence modifications, reducing risks of unintended or permanent genomic changes.

**Fig. 1:**
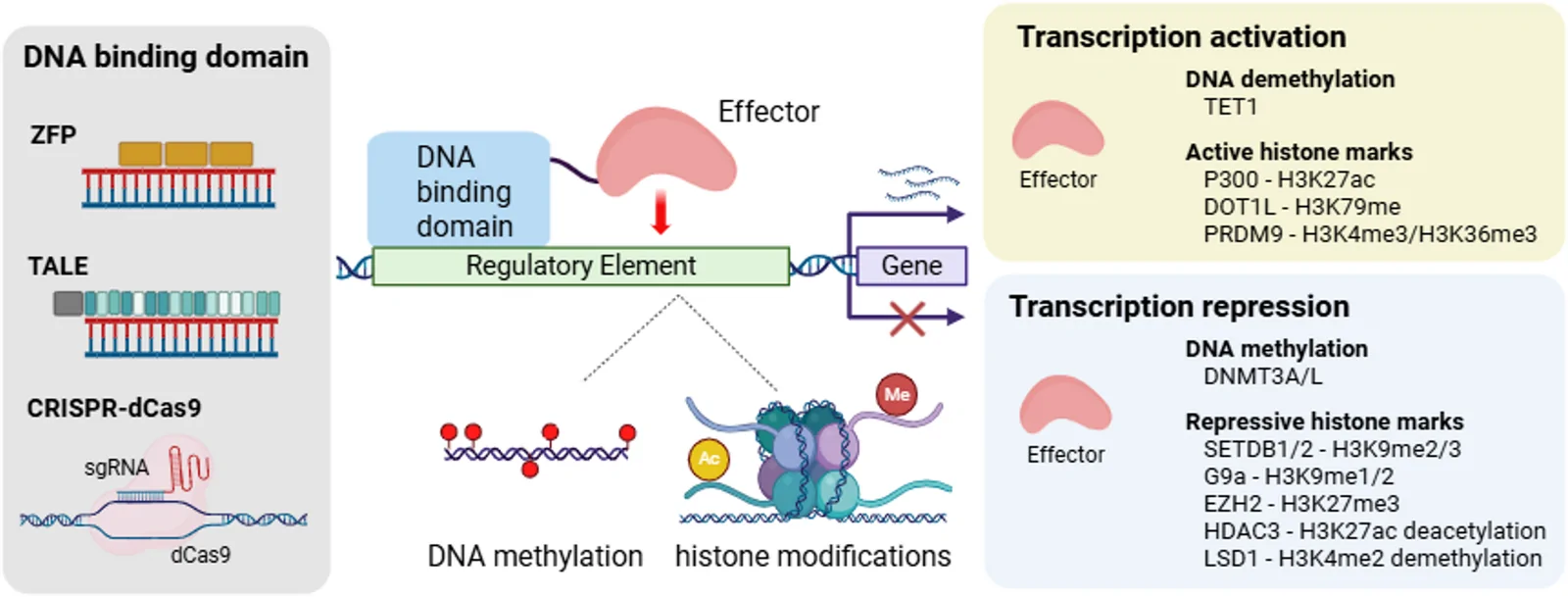
Modular structure of epigenome editors for transcriptional control. Schematics illustrating the modular components of an epigenome editor, including programmable DNA-binding domains and effector domains with activating or repressive functions (ZFP: Zinc finger protein, TALE: Transcription activator-like effector, CRISPR-dCas9: Clustered regularly interspaced short palindromic repeats-catalytically dead Cas9, sgRNA: single-guide RNA, ac: acetylation, me: methylation, me1: mono-methylation, me2: di-methylation, me3: tri-methylation)

Epigenome editing tools consist of a DNA-binding domain (DBD) and epigenetic effector module (Fig. 1) [11]. To date, three classes of DNA-targeting systems have been primarily adapted for a DBD: zinc finger proteins (ZFPs), transcription activator-like effectors (TALEs), and clustered regularly interspaced short palindromic repeats (CRISPR) systems [10, 11]. ZFPs and TALEs can be engineered to recognize specific DNA sequences, whereas the CRISPR system utilizes a catalytically dead Cas9 (dCas9) protein guided by a single-guide RNA (sgRNA), which enables flexible targeting of defined loci through simple sgRNA sequence design [12–14]. These DBDs can be fused to a variety of epigenetic effectors. Commonly used repressors include DNA methyltransferase 3A (DNMT3A), which catalyzes *de novo* DNA methylation, and the Krüppel-associated box (KRAB), which promotes heterochromatin formation by recruiting histone methyltransferases. Activators include the DNA demethylase ten-eleven translocation methylcytosine dioxygenase 1 (TET1), the histone acetyltransferase (HAT) p300, and the herpes simplex virus type 1 protein VP16 and its derivatives such as VP64 and VP160 [15–18]. Preclinical studies, both in vitro and in vivo, have demonstrated that epigenome editing can modulate the expression of disease-associated genes across monogenic disorders, cancers, neurological disorders, and chronic diseases. In this review, we provide a comprehensive overview of current advances in epigenome editing technologies and summarize the current status of their preclinical and clinical applications.

## Recent advances in epigenome editing

### Introduction to epigenome editing systems

CRISPR interference (CRISPRi), first developed in *Escherichia coli*, utilizes dCas9 to sterically hinder recruitment and assembly of the transcriptional machinery at promoters, thereby repressing target gene expression [19]. CRISPRi laid the foundation for dCas9-based epigenome editing systems in eukaryotes (Fig. 1) [17, 18, 20–23]. For example, dCas9 fused to KRAB (dCas9-KRAB) repressed endogenous *CD71* by 2-3-fold in HeLa cells compared to dCas9 alone [17, 20]. dCas9 fused to su(var)3–9, enhancer-of-zeste, and trithorax (SET) domain of G9A histone methyltransferase repressed *HER2* expression by 3.3-fold compared to the dCas9 alone control in HCT116, accompanied by a 13-fold increase in H3K9me3 levels at the promoter [24]. Interestingly, only the *N*-terminal G9A[SET]-dCas9 fusion demonstrated effective gene repression among the different fusion configurations (*C*-terminal-, *N*-, or dual-terminal), suggesting that the spatial configuration of the DBD and epigenetic effector module affects epigenetic editing efficiency.

Effector domains offer versatility in epigenome editing, enabling not only repression but also activation. When dCas9 is fused to a transactivation domain such as the tetrameric VP16 repeat construct VP64 (dCas9-VP64), it was shown to activate endogenous *MYOD* about 30-fold in HEK293T [17]. dCas9 fused with the p300 HAT core domain (dCas9-p300^core^) activated *IL1RN* in K562 cells with increased histone H3 lysine 27 acetylation (H3K27ac) at the target promoters, showing several-fold and 10- to 300-fold higher activation than dCas9-VP64 and full-length p300 fusions [17]. This demonstrates that the core domain alone is sufficient for effective epigenetic modification, as also seen with the dCas9-TET1 catalytic domain alone (dCas9-TET1CD), which reduces DNA methylation and reactivates genes [25].

### Epigenome editing with multiple epigenetic effectors

**Fig. 2:**
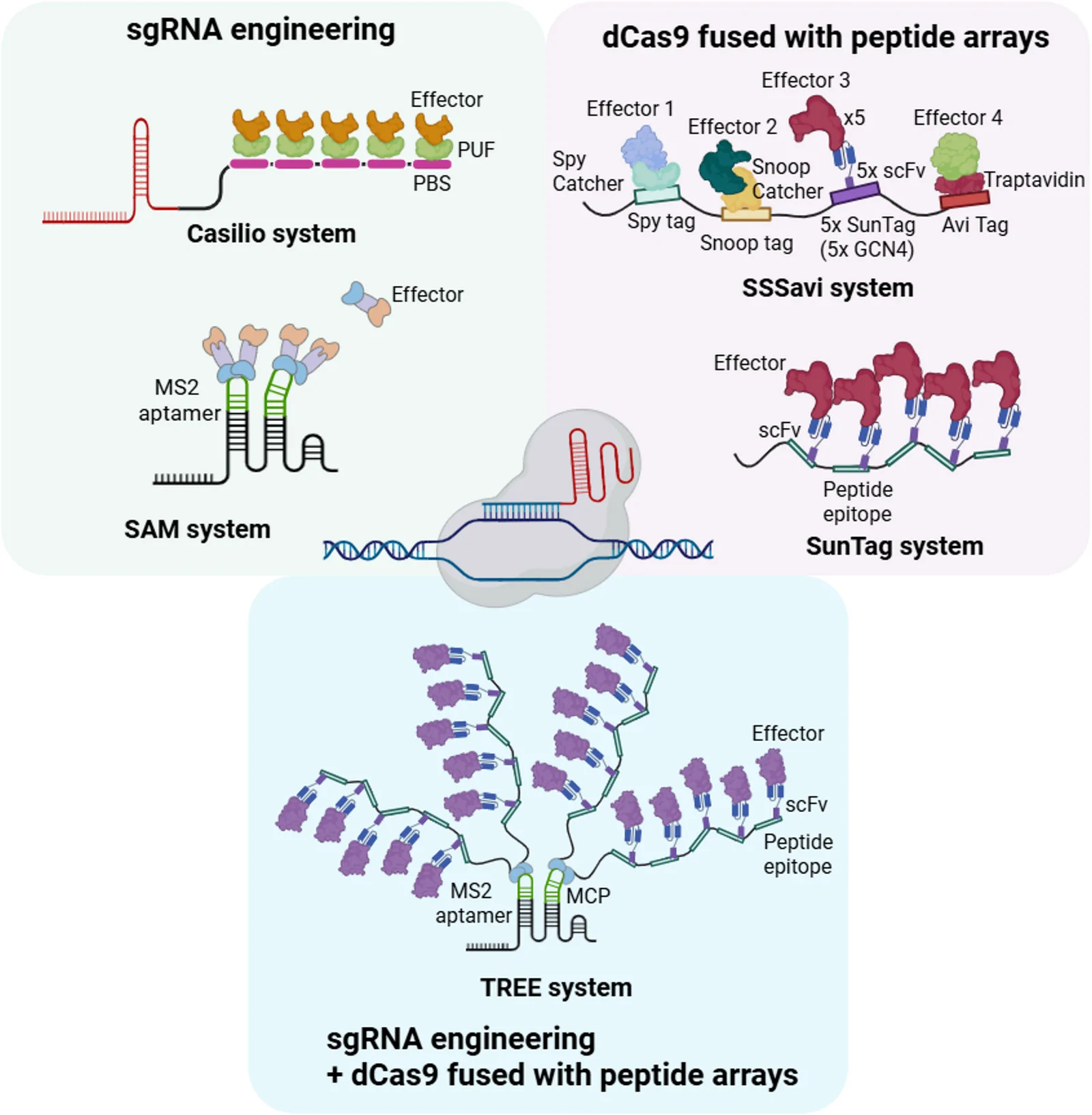
Multi-effector-based epigenome editors. Approaches for fusing multiple effector domains to a single dCas9. Casilio and synergistic activation mediator (SAM) systems use engineered single-guide RNAs (sgRNAs). SUperNova-Tagging (SunTag) system and SSSavi systems use peptide array-fused dCas9. The three-component repurposed technology for enhanced expression (TREE) system combines sgRNA engineering and dCas9 fused with peptide arrays (PBS: protein-binding sequences, PUF: Pumilio/FBF, scFv: single-chain variable fragment, MCP: MS2-coat protein)

Enhancing the efficiency of epigenome editing tools has driven the development of platforms that incorporate multiple epigenetic effector domains (Table 1). The approach involves using a dCas9 scaffold fused to multiple copies of a single epigenetic effector or fused to combinations of different epigenetic effectors (Fig. 2) [26–29]. One system includes SUperNova tagging system (SunTag) which induces protein multimerization through the interactions between a repeating peptide array and the single-chain variable fragment (scFv) [26]. In K562 cells, dCas9-SunTag-VP64, which carries a tandem array of GCN4 epitopes that can recruit up to ten scFv fused VP64 molecules, induced several tens of fold higher activation of endogenous *CXCR4* expression compared to dCas9-VP64.

**Table 1:** Comparison of the efficiency of multi-effector epigenome editors. Summary of the efficiency of multi-effector epigenome editors, highlighting differences in modulationured at the mRNA level. Reported fold changes in editing efficiency should not be directly compared across studies with different target loci, cell types, or experimental conditions efficiency across distinct effector architectures, target loci, and cellular contexts. Unless otherwise noted, each experiment tests an individual gene. Descriptions of epigenome editors consider the position of dCas9 fusions (Left: fused to the N-terminus of dCas9; Right: fused to the C-terminus of dCas9). All comparisons are made using the same sgRNA(s). All outcomes are measured at the mRNA level. Reported fold changes in editing efficiency should not be directly compared across studies with different target loci, cell types, or experimental conditions

Epigenome editor 1	Epigenome editor 2	Effect	Cell line	Gene target	Outcome	Ref
				(Promoter region is targeted, unless otherwise noted)	Fold change of activation or repression	
VP64-dCas9-VP64	dCas9-VP64	Activation	HEK293T	*ASCL1*	5.2	[30]
				*MYOD*	3.6	
				*NEUROD1*	2.1	
			Neuro-2a	*Neurog2*	3	
				*Hbb-bh1*	4.3	
dCas9-VP192	VP64-dCas9-VP64	Activation	HEK293T	*POU5F1*	3.7	[31]
				*SOX2*	2	
FOG[1–45]-dCas9-FOG[1–45]^a)^	dCas9-FOG[1–45]	Repression	HCT116	*HER2*	2	[24]
dCas9-SunTag-VP64 (10xGCN4 peptide array)	dCas9-VP64	Activation	K562	*CDKN1B*	2.6	[26]
dCas9-SunTag-p65HSF1 (10xGCN4 peptide array)	dCas9-SunTag-VP64 (10xGCN4 peptide array)	Activation	Neuro-2a	*Ascl1*	642	[32]
				*Neurog2*	37	
dCas9-Casilio-p65HSF1 (sgRNA containing 5xPBS)	dCas9-p65HSF1	Activation	HEK293T	*OCT4*	5.7	[27]
				*SOX2*	4	
	dCas9-Casilio-p65HSF1 (sgRNA containing 1xPBS)			*OCT4*	Several hundreds	
				*SOX2*	8	
dCas9-Casilio-CBPHAT^b)^ (sgRNA containing 5xPBS)	dCas9-CBPHAT			*OCT4*	No significant change	
				*OCT4* proximal enhancer	6	
				*OCT4* distal enhancer	Several hundreds	
dCas9-SSSavi (SpyTag-p65HSF1, SnoopTag-p65HSF1, 5xSunTag-p65HSF1, AviTag-p65HSF1)	dCas9-VP64	Activation	HEK293T	*KL*	Several hundreds	[33]
				*EPCAM*	13	
				*PACC1*	2	
	dCas9-p65HSF1			*KL*	Several hundreds	
				*EPCAM*	12	
				*PACC1*	2	
	dCas9-VPR			*KL*	Several tens	
				*EPCAM*	3	
				*PACC1*	0.8	
	dCas9-SunTag-VP64 (5xGCN4 peptide array)			*KL*	Several tens	
				*EPCAM*	8	
				*PACC1*	2	
	dCas9-SunTag-p65HSF1 (5xGCN4 peptide array)			*KL*	2.5	
				*EPCAM*	No significant change	
				*PACC1*	0.8	
dCas9-VP64-p65-Rta (dCas9-VPR)	dCas9-VP64	Activation	HEK293T	*MIAT*	40	[34]
				*NEUROD1*	29	
				*ASCL1*	42	
				*RHOXF2*	22	
				*TTN*	317	
				*ACTC1*	94	
			iPSC	*NGN2*	10	
				*NEUROD1*	18	
dCas9-KRAB-MeCP2	dCas9-KRAB	Repression	HEK293T	*CANX*	2.6	[35]
				*CXCR4*	7.7	
				*CHK1*	3.3	
				*SEL1L*	2.4	
				*ARPC2*	2	
				*MAPK3*	2.3	
				*BRCA1*	1.1	
dCas9-VP64 + MCP-p65HSF1 + sgRNA containing 2xMS2 stem loops	dCas9-VP64	Activation	HEK293FT	*VEGFA*	At least 2-fold and up to several thousand-fold across all tested genes	[36]
				*HBG1*		
				*TERT*		
				*IL1B*		
				*IL1R2*		
				*MYC*		
				*ZFP42*		
				*LIN28A*		
				*SOX2*		
				*NANOG*		
				*KLF4*		
				*POU5F1*		
dCas9-VP64 + MCP-4xGCN4 peptide array + scFv-p65HSF1 + sgRNA containing 2xMS2 stem loops	dCas9-VP64	Activation	MIA-PaCa2	*CDH1*	200	[37]
	dCas9-VP64 + MCP-p65HSF + sgRNA containing 2xMS2 stem loops				2.5	
dCas9-VP64 + MCP-4xGCN4 peptide array + scFv-VPR + sgRNA containing 2xMS2 stem loops	dCas9-VP64	Activation	HEK293T	*RANKL*	11	
	dCas9-VP64 + MCP-VPR + sgRNA containing 2xMS2 stem loops				Several tens	
dCas9-p300[Core] + MCP-VP64 + sgRNA containing 2xMS2 stem loops	dCas9-VP64	Activation	HEK293T	*MYOD enhancer*	11	[38]
				*HS2 enhancer*	*HBE1*: 11	
					*HBG1/2*: 72	
					*HBB*: 4.6	
	dCas9-p300[Core]			*MYOD enhancer*	1.9	
				*HS2 enhancer*	*HBE1*: 2.9	
					*HBG1/2*: 2.4	
					*HBB*: 1.8	
dCas9-KRAB + MCP-LSD1 + sgRNA containing 2xMS2 stem loops	dCas9-KRAB	Repression	HEK293T	*HS2 enhancer*	*HBE1*: 3	
					*HBG1/2*: 5	
					*HBB*: 1.6	
	dCas9-LSD1				*HBE1*: 2.5	
					*HBG1/2*: 2.6	
					*HBB*: 3.5	
dCas9-SSSavi (SpyTag-DNMT3A, 5xSunTag-KRAB, AviTag-EZH2)	dCas9-SSSavi (SpyTag-KRAB, 5xSunTag -EZH2, AviTag-DNMT3A)	Repression	HEK293T	*B2M*	1.6	[33]
				*RBM3*	1.7	
				*HINT1*	2.2	

a) FOG[1–45]: N-terminal 45 residues of Friend of GATA1 (FOG)

b) CBHAT: Histone acetyltransferase domain of CRE-binding protein

dCas9-synergistic activation mediator (SAM) utilizes an engineered sgRNA containing two or three MS2 hairpin repeats. MS2 coat protein (MCP)-fused epigenetic effectors are recruited to a single dCas9 via the MCP-MS2 interaction [36]. For example, dCas9-VP64-MS2 with MCP-p65 increases *ASCL1* expression in HEK293FT cells by several tens of fold compared with dCas9-VP64.

A Three-component Repurposed technology for Enhanced Expression (TREE) system integrates the SunTag and SAM platforms [37]. In this design, multiple MCPs fused to GCN4 epitope arrays are recruited to a single dCas9 via MS2 stem loops within engineered sgRNAs. Subsequently, multiple scFv-fused effector proteins are recruited to the GCN4 epitope arrays on each MCP. This branched architecture enables robust transcriptional activation with a single dCas9-based epigenome editor.

Another system is dCas9-Casilio, which uses an engineered sgRNA containing multiple 8-mer sequences recognized by the Pumilio/FBF (PUF) RNA-binding domain [27]. These repeats recruit multiple PUF-fused effectors to a single dCas9.

Recently, a novel combinatorial recruitment system termed Spy-Snoop-Sun-Avi (SSSavi) was developed, in which four protein–protein interaction domains, SpyTag, SnoopTag, SunTag, and AviTag, are sequentially fused to a dCas9 scaffold [33]. Each tag recruits a corresponding binding partner, referred to as a “catcher”: SpyCatcher, SnoopCatcher, αGCN4 and Traptavidin, respectively. By conjugating effector proteins to these catchers, up to four distinct effectors can be recruited to a single dCas9 in a spatially controlled order. The SSSavi system has been used to investigate how the spatial arrangement of effectors affects editing efficiency. The combination of KRAB, DNMT3A, and enhancer of zeste homolog 2 (EZH2) showed different transcriptional repression effects depending on their spatial order. The strongest editing efficiency was observed in the sequence of DNMT3A, KRAB, and then EZH2 while the sequence of KRAB, EZH2, and then DNMT3A showed little to no repression. This result may reflect the requirement for optimal positioning of effectors relative to their targets. For example, DNMT3A preferentially methylates linker DNA, thereby constraining the chromatin contexts in which productive DNA methylation can occur [39, 40]. Similarly, EZH2 activity is influenced by dinucleosome architecture and requires specific chromatin organization for efficient deposition of H3K27me3 [41]. Thus, thorough evaluation of effector architecture, including effector combinations and their relative spatial arrangements is required for the development of optimal epigenome editors, as these factors can substantially affect editing outcomes.

Another advantage of using multiple effectors is to allow for studying synergistic or antagonistic interactions between epigenetic effectors [35, 38, 42–44]. Using a dCas9-based MS2-MCP recruitment system, over 50,000 bivalent effector pairs are systematically evaluated for their regulatory effects on the *CD81* membrane protein gene in K562 cells. In the pairwise assay, two effectors were physically fused in tandem to the C-terminus of MCP (MCP-linker-effector1-linker-effector2). Notably, HAT members such as lysine acetyltransferase 5 (KAT5) and p300 completely neutralized HDAC-driven repression through the antagonistic interaction. The KRAB domain of ZNF10 exhibited the most potent synergistic repressive effect when paired with lethal (3) malignant brain tumor-like protein 3 (L3MBTL3), polycomb repressive complex 2–associated ligand-dependent nuclear receptor corepressor isoform 1 (PALI1) or elongin BC and polycomb repressive complex 2-associated protein (EPOP). These combinations markedly strengthened *CD81* repression compared with GFP-KRAB control.

### Inducible epigenome editing systems

The development of light- or chemically inducible epigenome editing technologies has enabled the spatiotemporal control of gene expression and minimized off-target effects. This controlled regulation is crucial for recapitulating the transient and context-dependent nature of transcriptional regulation in living cells.

**Fig. 3:**
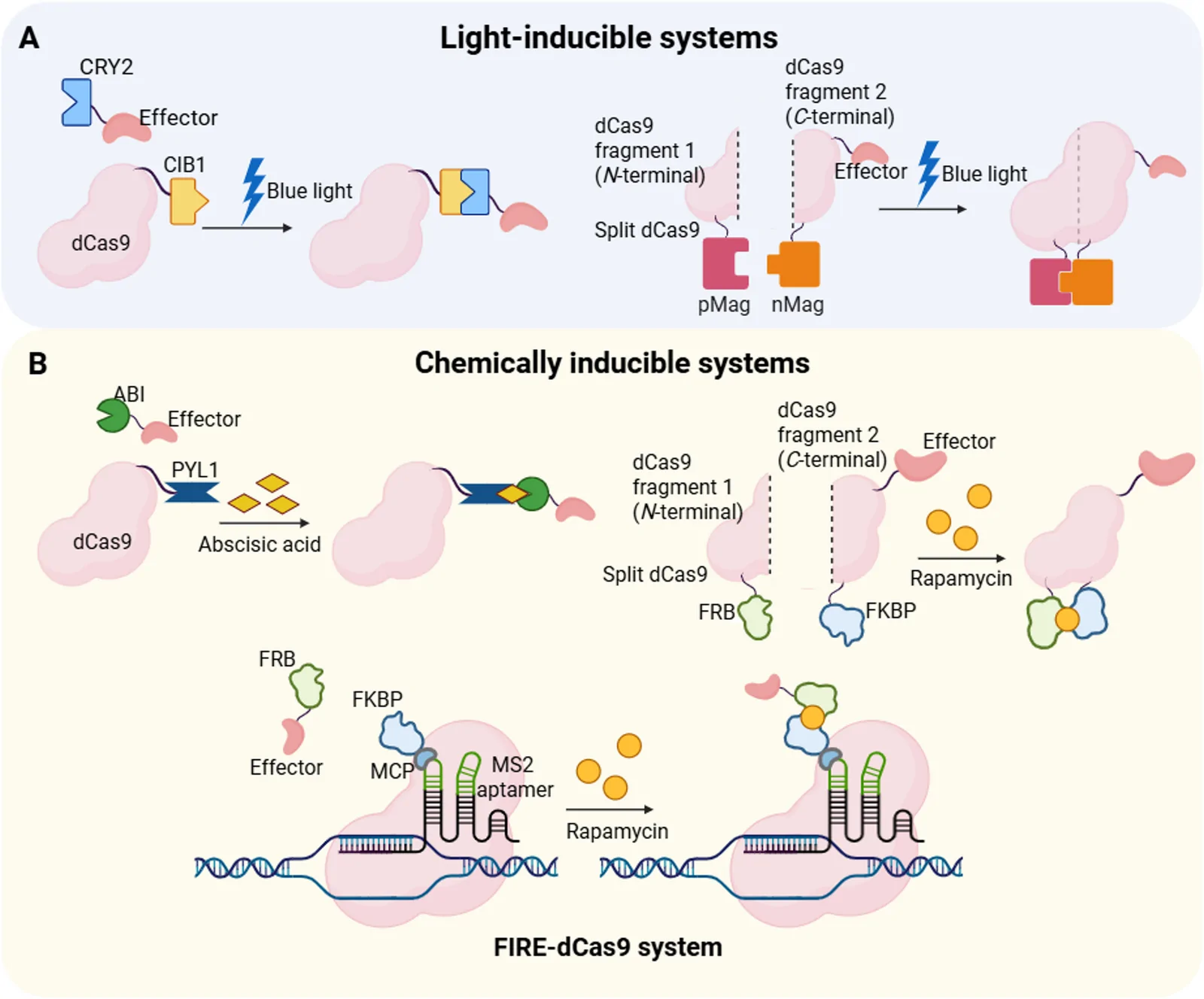
Inducible epigenome editors. (**A**) Examples of light-inducible epigenome editors. Light-inducible epigenome editors based on the interaction between cryptochrome 2 (CRY2) and cryptochrome-interacting basic helix-loop-helix 1(CIB1) (left) and on the Magnet protein (right). (**B**) Examples of chemically inducible epigenome editors. Chemically inducible epigenome editors based on the abscisic acid-mediated pyrabactin resistance-like 1 (PYL1)-abscisic acid insensitive interaction (top left) and rapamycin-mediated FK506-binding protein (FKBP)-FKBP-rapamycin-binding domain (FRB) interaction (top right and bottom) (FIRE-dCas9: FKBP-FRB inducible recruitment for epigenome editing by dCas9, pMag: positive Magnet protein, nMag: negative Magnet protein)

Light-inducible epigenome editing exploits photosensitive proteins that dimerize upon light exposure. For example, cryptochrome 2 (CRY2) and its partner cryptochrome-interacting basic helix-loop-helix 1 (CIB1) reversibly heterodimerize under blue light on a sub-second timescale, providing a platform for the rapid light-controlled molecular interaction **(**Fig. 3A**)** [45, 46]. These photosensitive proteins have been engineered to adapt to an epigenome editing system [47]. CRY2 was truncated to a CRY2 fragment only containing the photolyase homology region (CRY2PHR) to achieve stronger light-mediated transcriptional activation. A truncated CIB1 (trCIB1) was generated by removing its intrinsic TF-like domain, which otherwise causes background activation in the absence of light. In HEK293T cells, dCas9-trCIB1 and CRY2PHR-p65 enabled rapid and light-dependent activation of *ASCL1* expression, resulting in a 51-fold higher transcription activation compared to the dark control [48]. A 10-fold increase in *ASCL1* expression was achieved after only 3 h of irradiation. The activation was reversible, as *ASCL1* mRNA levels returned to baseline within 18 h of dark incubation and could be re-induced upon subsequent light exposure. In another study, one dCas9-trCIB1 targets *Zfp462* promoter, while the other targets a putative *Klf4* enhancer located approximately 500 kb away from *Zfp462* promoter [49]. The light-induced chromatin loop increased *Zfp462* expression approximately 1.2-fold relative to the dark control. These results indicate that light-inducible *de novo* chromatin loop formation can selectively enhance target gene expression.

Some light-inducible systems are based on split-dCas9 in which the dCas9 protein is split into two fragments, *N*-terminal and *C*-terminal fragments **(**Fig. 3A**)** [50, 51]. Each fragment is fused to the positive Magnet protein (pMag) and the optimized variant of negative Magnet protein (nMagHigh1), respectively. These paired Magnet proteins undergo blue light-inducible heterodimerization. This Magnet-based split-dCas9 system targeting *ASCL1* exhibited several tens-fold higher gene induction compared with the CRY2-CIB1 based systems in HEK293T cells [51]. Moreover, the increased *ASCL1* expression was maintained for up to 36 h after blue light was turned off, whereas the CRY2-CIB1 based system returned to baseline expression [51]. Similarly, split-dCas9 fused to VP64 and MCP-p65-HSF1 induced a higher increase in *NEUROD1* expression relative to the CRY2-CIB1 based system in human induced pluripotent stem cells [51]. Correspondingly, neuronal differentiation was confirmed by the appearance of the neuronal marker TUJ1, whereas the CRY2-CIB1 based system failed to do so.

In parallel, chemically inducible epigenome editing tools have been developed. For example, dCas9-based systems utilize a rapamycin-inducible interaction between the FK506-binding protein (FKBP) and the FKBP-rapamycin-binding domain (FRB). In the Split-dCas9 system, each dCas9 fragment is conjugated with FKBP and FRB **(**Fig. 3B**)** [52]. The *N*-terminal fragment of dCas9 fused to FRB is engineered with two nuclear export signals to localize predominantly in the cytoplasm, whereas the *C*-terminal fragment of dCas9 fused to FKBP contains two nuclear localization signals (NLSs) for nuclear localization. This spatial separation minimizes spontaneous reconstitution prior to rapamycin-induced dimerization. Upon rapamycin treatment, the fragments are brought together to reconstitute functional epigenome editors. Following 24 h of rapamycin treatment, this rapamycin inducible split-dCas9 system activated *ASCL1* expression to the level comparable to that of full-length dCas9 [52]. In the FKBP-FRB inducible recruitment for epigenome editing by dCas9 (FIRE-dCas9) system, a MS2-modified sgRNA recruits FKBP-MCP adaptors, and it dimerizes with FRB-fused effectors only in the presence of rapamycin **(**Fig. 3B**)**. The FIRE-dCas9 fused to the Brahma-associated factor (BAF) complex activated gene expression within an hour after rapamycin induction, culminating in up to a 20-fold increase by 48 h [9]. FK506 treatment reversed these rapamycin-induced effects within 24 h.

Another chemically inducible epigenome editing system employs abscisic acid (ABA) as a ligand, a plant phytohormone that induces the dimerization between the abscisic acid insensitive protein and pyrabactin resistance-like 1 (PYL1) protein (Fig. 3B). dCas9-PYL and ABI-p300 HAT induced a time-dependent increase in H3K27ac at the *IL1RN* gene promoter in HEK293T cells [53]. Upon ABA treatment, H3K27ac levels began to rise in 1 h. *IL1RN* expression increased after 4 h and reached its maximum level at 24 h. Following ABA withdrawal, both H3K27ac and *IL1RN* rapidly returned to baseline levels within 1 h. Interestingly, despite similar maximum *IL1RN* expression levels, 48-hour ABA exposure resulted in a slower decline of H3K27ac and *IL1RN* expression after ABA washout compared with the 24-hour treatment group. In another study, dCas9-ABI and PYL1 fused with coilin, a scaffold protein of Cajal bodies induced three-dimensional chromatin reorganization [54]. The targeted colocalization of a Chr3 locus with Cajal bodies via ABA treatment for 4 days resulted in the simultaneous repression of multiple distantly located genes. Specifically, expression of *ACAP2*, located 35 kb upstream of the sgRNA-targeted loci, was reduced by 3.3-fold, expression of *PPP1R2*, located 36 kb downstream, was decreased by 7.7-fold, and expression of *TFRC*, 575 kb downstream, was reduced by 2.8-fold. The modulation of chromatin architecture enables the regulation of multiple distal genes with a single sgRNA and provides a novel tool to dissect the causal relationship between three-dimensional chromatin architecture and transcriptional regulation.

Despite their temporal control, inducible epigenome editing systems have remaining challenges including basal leakiness, potential off-target effects of chemical inducers, and limited in vivo applicability due to poor light penetration, and the delivery requirements of multi-component architectures.

## Epigenome editing applications for functional enhancer studies

Enhancers are attractive targets for epigenome editing due to their gene-, cell-type- and time-specific control of gene expression. Epigenome editing techniques have been increasingly applied to elucidate enhancer function. For example, dCas9 fused to lysine-specific histone demethylase 1 (dCas9-LSD1) was used to identify functional enhancer of *Tbx3*, a key regulator of pluripotency in mouse embryonic stem cells (mESCs) [55]. Screening of candidate enhancers revealed a regulatory element located approximately 10 kb upstream of the *Tbx3* promoter, whose repression reduced H3K4me2 and H3K27ac levels and downregulated *Tbx3* expression. The functional enhancer of *HER2* was identified using a similar approach. Screening of 433 DNase I hypersensitive sites (DHSs) surrounding *HER2* locus using dCas9-KRAB and dCas9-p300 identified regulatory elements within the first intron of *HER2* and *GRB7* that modulate *HER2* expression [56]. These studies demonstrate the utility of epigenome editing for the systematic identification of functional gene regulatory elements.

Beyond identifying individual enhancers, epigenome editing tools can be applied to elucidate hierarchical regulatory functions of multiple enhancers targeting the same gene. Ten estrogen receptor α binding sites (ERBSs) were investigated using a pool of 42 sgRNAs and four concatenated mSin3 interaction domains (SID4X) fused to N-terminal of dCas9-KRAB (SID4X-dCas9-KRAB) in Ishikawa cells [57]. This study revealed “predominant” and “supportive” enhancers. The predominant ERBS alone markedly reduced gene expression, whereas the supportive ERBS alone showed a much smaller inhibitory effect and produced additional transcriptional repression only when co-inhibited with the predominant ERBS. These findings indicate that each enhancer contributes unequally, forming a hierarchical enhancer structure.

Epigenome editing can also be used to investigate lineage-specific enhancer functions in vivo. Hematopoietic stem/progenitor cells (HSPCs) expressing doxycycline-inducible dCas9-KRAB were retrovirally transduced with pooled sgRNA-MS2 constructs designed for targeting fourteen enhancers of key TF genes, including *Cebpa*,* Spi1*,* Gata1*,* Gata2* and *Runx1*. The transduced cells were co-expressed with MCP-LSD1 and were subsequently transplanted into the bone marrow of lethally irradiated recipient mice to assay lineage differentiation outcomes [38]. The enhancers located at 8 kb downstream of *Cebpa* transcription start site (TSS), 37 kb downstream of *Cebpa* TSS, and 9.5 kb downstream of *Gata2* TSS were essential for myelopoiesis, whereas *Spi1* enhancer was required for both myeloid and B-cell differentiation. In contrast, *Gata1* and *Runx1* enhancers, despite their accessibility and active histone marks in HSPCs, were dispensable for lineage commitment.

Since enhancer activity is often pleiotropic, redundant, and context-dependent, careful assessment of epigenome editing-based enhancer studies is required. A single enhancer may regulate multiple genes and cellular phenotypes [58–60]. Perturbation of an individual enhancer may not produce a measurable effect due to functional redundancy among enhancers [61, 62]. Enhancer activity can also vary across cell types and developmental states [63–65]. These properties can complicate both mechanistic interpretation and therapeutic targeting, highlighting the need for caution when translating enhancer perturbation findings across biological contexts.

## Epigenome editing applications for disease control

Restoring the physiological expression levels of disease-associated genes is a promising therapeutic strategy. Epigenome editing has become increasingly explored as a therapeutic strategy for a wide range of diseases, from monogenic disorders to chronic diseases. While some studies remain at the proof-of-concept stage with efficacy demonstrated only in preclinical models, others have advanced toward clinical translation. In particular, epigenome editing for neurodegenerative disorders and neurodevelopmental disorders is still largely in early development. However, epigenome editing therapeutics for cancer, hepatitis B, and facioscapulohumeral muscular dystrophy have progressed to clinical trials and those for Duchenne muscular dystrophy and hypercholesterolemia have shown promising results in nonhuman primates. In this review, we discuss a range of epigenome editing systems, with a focus on their ability to suppress pathological phenotypes across various disease models (Table 2).

**Table 2:** Application of epigenome editors to various diseases. Overview of reported applications of epigenome editors across diverse disease categories. For each disease model, the target gene, target region, DNA-binding domain, effector domain, regulatory outcome, and key functional findings observed in vitro and in vivo are summarized. Outcome symbols are defined as follows: ↑, increased; ↓, decreased; ND, not determined

Disease Category	Target Disease	Target Gene	Target Region	DNA-binding Domain	Effector domain	Molecular outcomes	Pathway-level outcomes		Phenotypic outcomes	Ref
Monogenic disorder	Cystic Fibrosis	*CFTR*	Promoter	dCas9	VPR	(in vitro) *CFTR* mRNA ↑ CFTR protein ↑	(in vitro & in vivo) CFTR chloride channel activity ↑		(in vitro & in vivo) chloride transport ↑	[66]
Monogenic disorder	Beckwith–Wiedemann syndrome	*H19*,* IGF2*	Imprinting control region 1	dCas9 (SunTag)	TET1CD	(in vitro) *H19* mRNA ↑ *IGF2* mRNA ↓	ND		ND	[67]
Monogenic disorder	Marfan Syndrome	*FBN1*	CpG island shore	dCas9	TET1CD	(in vitro) *FBN1* mRNA ↑	ND		ND	[68]
Monogenic disorder	Retinitis Pigmentosa	*Opn1mw*	Promoter	dCas9	VPR	(in vitro) *Opn1mw* mRNA ↑ (in vivo) M-opsin protein ↑	(in vivo) retinal degeneration ↓		(in vivo) outer nuclear layer thickness ↑ retinal function ↑	[69]
Monogenic disorder	Hutchinson–Gilford progeria syndrome	*Oct4*	Promoter	dCas9	VPR	(in vitro) *Oct4* mRNA ↑ progerin ↓ (in vivo) *p16*/*p21*/*IL-6* ↓	ND		(in vivo) aortic integrity ↑ weight loss ↓ lifespan ↑	[70]
Cancer	Aggressive cancers	*PTEN*	Promoter	dCas9	VPR	(in vitro) *PTEN* mRNA/protein ↑		(in vitro) AKT/mTOR/MAPK^a)b)c)^ signaling ↓	(in vitro) migration ↓ drug sensitivity ↑ (dabrafenib, dactolisib)	[71]
Cancer	Gastric cancer	*REPRIMO*	Promoter	dCas9	VPR (SAM^d)^ system)	(in vitro) Ki67 ↓ caspase-3 ↑		ND	(in vitro) viability ↓ proliferation ↓ apoptosis ↑	[72]
Cancer	Lung cancer	*SERPINB5*	Promoter	dCas9	VPR (SAM^d)^ system)	(in vitro) Ki67 ↓ caspase-3 ↑		ND	(in vitro) viability ↓ proliferation ↓ apoptosis ↑	[72]
Cancer	Lung and Kidney cancer	*ZAR1*	Promoter	dCas9	TET1CD, p300, VP160	ND		ND	(in vitro) proliferation ↓ G1/S ↑ G2/M ↓	[73]
Cancer	Breast cancer	*BRCA1*	Promoter	dCas9	TET1CD	ND		ND	(in vitro) proliferation ↓ survival ↓	[74]
Cancer	Multiple tumor models	*miR-524*	Enhancer	dCas9	VP64	(in vitro) *miR-524* ↑ Smad2/Hes1/Tead1 protein ↓		(in vivo) TGF-β/Notch pathways ↓	(in vivo) tumor growth ↓	[75]
Cancer	Colon cancer	*SARI*	Promoter	dCas9	TET1CD	(in vitro) *SARI* mRNA/protein ↑		ND	(in vitro & in vivo) angiogenesis ↓ proliferation ↓ apoptosis ↑	[76]
Cancer	Oesophageal adenocarcinoma	*JUP*,* MYBL2*,* CCNE1*	Enhancer	dCas9	KRAB	(in vitro) *JUP*/*MYBL2*/*CCNE1* mRNA ↓		ND	(in vitro) survival ↓	[77]
Cancer	Hepatocellular carcinoma	*HHIP*,* MT1M*,* PZP*,* TTC36*	Promoter	dCas9	VPR (SAM^d)^ system)	(in vitro) *HHIP/MT1M/PZP/TTC36* mRNA ↑		ND	(in vitro) proliferation ↓ viability ↓ migration ↓	[78]
Cancer	Hepatocellular carcinoma	*MYC*	Promoter, Enhancer anchoring site	ZFP	MQ1, KRAB	(in vitro) *MYC* mRNA/protein ↓		ND	(in vitro & in vivo) proliferation ↓ antitumor activity ↑	[79]
Cancer	Anti-VEGF^e)^ antibody–resistant ovarian cancer	*BARD1*	Promoter	dCas9	TET1CD	(in vivo) *BARD1* mRNA/expression ↑		(in vivo) Anti-VEGF^e)^ antibody resistance/angiogenesis pathway ↓	(in vivo) tumor burden ↓ Anti-VEGF^e)^ antibody resistance ↓ stable body weight	[80]
Cancer	Thyroid cancer	*TERT*	Promoter	dCas9	TET1CD	(in vitro) *TERT* mRNA ↓		ND	(in vitro) apoptosis ↑ cell growth ↓	[81]
Cancer	Breast cancer	*miR-200c*	Promoter	dCas9	TET1CD	(in vitro) E-cadherin ↑		(in vitro) epithelial-to-mesenchymal transition markers ↓	(in vitro) viability ↓ apoptosis ↑	[82]
Cancer	Cervical cancer	*EphA7*	Promoter	dCas9	TET1CD	(in vitro) *EphA7* mRNA/protein ↑ Bax ↑ Bcl-2 ↓ caspase-3 ↑		(in vitro) PI3K/AKT^f)^ signaling ↓ epithelial-mesenchymal transition reversal	(in vitro) proliferation ↓ invasion ↓ apoptosis ↑ chemo sensitivity ↑	[83]
Cancer	Solid tumor, Hematologic malignancy	*RASA2*	Promoter	dCas9	DNMT3A-DNMT3L-KRAB	ND		ND	(in vitro) CAR-T^g)^ killing/persistence ↑ (in vivo) tumor burden ↓ CAR-T durability ↑ survival ↑	[84]
Cancer	Glioblastoma	*MGMT*	Promoter	dCas9	DNMT3A-DNMT3L-KRAB	ND		ND	(in vitro) temozolomide, lomustine sensitivity ↑ apoptosis ↑ (in vivo) tumor regression ↑ time-to-regression ↓	[85]
Neurodegenerative disorder	Alzheimer’s disease	*Dlg4*	Promoter	ZFP	VP64	(in vitro) *Dlg4* mRNA ↑ PSD95 protein ↑		ND	(in vivo) hippocampal memory ↑	[86]
Neurodegenerative disorder	Age-related memory impairment	*Per1*	Promoter	dCas9	VP64 (SAM^d)^ system)	(in vitro) *Per1* mRNA ↑		(in vivo) synaptic plasticity normalization	(in vivo) object location memory ↑	[87]
Neurodegenerative disorder	Alzheimer’s disease	*Syt1*	Promoter	dCas9	KRAB	(in vitro, in vivo) *Syt1* mRNA ↓		(in vitro) neurotransmitter release ↓ excitatory/inhibitory balance modulated	(in vivo) anxiety-like behavior ↓	[88]
Neurodegenerative disorder	Late onset Alzheimer’s disease	*ApoE*	Promoter	dSaCas9	KRAB, MeCP2-TRD	(in vivo) ApoE protein ↓		ND	ND	[89]
Neurodegenerative disorder	Alzheimer’s disease	*Gad1*	Promoter	dCas9	p300	(in vitro & in vivo) *Gad1* mRNA ↑		(in vivo) γ-aminobutyric acid-mediated inhibitory postsynaptic currents ↑ synaptic inhibition ↑	(in vivo) spatial memory ↑	[90]
Neurodegenerative disorder	Alzheimer’s disease	*Bace1*	Promoter	dCas9	DNMT3A	(in vitro & in vivo) *Bace1* mRNA/protein ↓		(in vivo) amyloid pathology ↓	(in vivo) recognition memory ↑	[91]
Neurodegenerative disorder	Alzheimer’s disease	*App*	Promoter	dCas9	DNMT3A	(in vitro & in vivo) *App* mRNA/protein ↓ amyloid β 42/40 ↓		(in vivo) synaptic pathology ↓	(in vitro) neuronal survival ↑ neurite length ↑ (in vivo) memory performance ↑	[92]
Neurodegenerative disorder	Alzheimer’s disease	*Ctsd*	Promoter	dCas9	TET1CD	(in vitro) *Ctsd* mRNA/protein ↑ (in vitro & in vivo) amyloid β 42/40 ↓		(in vivo) synaptic pathology ↓	(in vitro) neuronal survival ↑ neurite length ↑ (in vivo) memory performance ↑	[93]
Neurodegenerative disorder	Parkinson’s disease	*SNCA*	Promoter	dSaCas9	2x KRAB	(in vitro) *SNCA* mRNA ↓ α-synuclein protein ↓		(in vitro) oxidative stress ↓ mitochondrial DNA damage ↓	ND	[94]
Neurodegenerative disorder	Parkinson’s disease	*SNCA*	CpG island in intron 1	dCas9	DNMT3A	(in vitro) *SNCA* mRNA ↓ α-synuclein protein ↓		(in vivo) VMAT2^h)^ function ↑	(in vitro) 1-methyl-4-phenylpyridinium induced neuronal toxicity ↓ neuronal viability ↑ (in vivo) dopaminergic apoptosis ↓ motor behavior ↑	[95]
Neurodegenerative disorder	Huntington disease	*mHTT*	CAG repeat in exon 1	ZFP	KRAB	(in vitro & in vivo) m*HTT* mRNA/protein ↓ (in vivo) medium spiny neuron markers ↑		ND	(in vivo) electrophysiology ↑ motor function ↑ anxiety-like behavior ↓	[96]
Neurodegenerative disorder	Prion disease	*Prnp*	Promoter	ZFP	H3 tail, DNMT3L	(in vitro & in vivo) *Prnp* mRNA ↓		ND	ND	[97]
Neurodevelopmental disorders	Fragile X Syndrome	*FMR1*	promoter	dCas9	TET1CD	(in vitro) *FMR1* mRNA ↑ FMRP protein ↑		ND	(in vitro & in vivo) electrophysiology normalization	[98]
Neurodevelopmental disorders	Dravet Syndrome	*Scn1a*	Promoter	dCas9	VP64	(in vitro, in vivo) *Scn1a* mRNA ↑		(in vitro) voltage-gated sodium channel ↑ interneuron firing ↑	(in vivo) febrile seizures ↓	[99]
Neurodevelopmental disorders	Rett syndrome	*MeCP2*	Promoter	dCas9	TET1CD, CTCF	(in vitro) *MeCP2* mRNA ↑		ND	(in vitro) soma size ↑ electrophysiology defects reversal	[100]
Neurodevelopmental disorders	Angelman syndrome	*UBE3A-ATS*	Snrpn imprinting center	dCas9 (SunTag)	DNMT3A-DNMT3L	(in vitro & in vivo) *Ube3a-ATS* mRNA ↓ paternal Ube3a mRNA ↑ Snrpn mRNA ↓		ND	(in vitro) soma size ↑ electrophysiology defects reversal	[101]
Neurodevelopmental disorders	Prader-Willi syndrome	*SNRPN*	Promoter	dCas9	TET1CD	(in vitro) neuron-specific Prader–Willi syndrome-related genes ↑ (*MAGEL2*,* NDN*)		ND	ND	[102]
Neurodevelopmental disorders	Prader-Willi syndrome	*SNRPN*	Imprinting control region	dCas9 (SunTag)	TET1CD	(in vitro) imprinted gene mRNA ↑		ND	(in vitro) neuronal activity ↑	[103]
Neurodevelopmental disorder	Intellectual disability	*Sema6a*	Promoter	dCas9 (SunTag)	C11orf46	(in vitro & in vivo) *Sema6a* mRNA ↓		ND	(in vivo) transcallosal connectivity ↑	[104]
Neurodevelopmental disorder	Adolescent alcohol exposure	*Arc*	Enhancer	dCas9	p300	(in vivo) *Arc* mRNA ↑		ND	(in vivo) anxiety-like behavior ↓ alcohol consumption ↓	[105]
Mental Disorders	Mood and anxiety disorders	*Ttr*	Promoter	dCas9	DNMT3A-DNMT3L	ND		ND	(in vivo) stress resilience ↑ depressive behavior ↓	[106]
Neuromuscular disorders	Duchenne muscular dystrophy	*Utrophin*	Promoter	dCas9	VP64	(in vitro) *Utrophin* mRNA/protein ↑ DAG1 localization restored		ND	(in vitro) calcium handling ↑ electrophysiology ↑	[107]
Neuromuscular disorders	Duchenne muscular dystrophy	*Utrophin*	Promoter	dCasMINI	VPR	(in vitro & in vivo) *Utrophin* mRNA/protein ↑		ND	(in vivo) skeletal/cardiac muscle ↑	[108]
Neuromuscular disorders	Congenital muscular dystrophy type 1A	*Lama1*	Promoter	dSaCas9	2x VP64	(in vivo & in vitro) *Lama1* mRNA/protein ↑ (in vivo) laminin-α1 localization ↑		ND	(in vivo) muscle architecture restored fibrosis/paralysis ↓	[109]
Neuromuscular disorders	Facio-scapulohumeral muscular dystrophy	*DUX4*	Exon 3	dCas9	KRAB	(in vitro) *DUX4* mRNA ↓ DUX4 target genes *TRIM43*/*ZSCAN4* mRNA ↓		ND	ND	[110]
Chronic Disease	Obesity	*Sim1*,* Mc4r*	Promoter, Enhancer	dCas9	VP64	(in vivo) *Sim1*/*Mc4r* mRNA ↑		ND	(in vivo) food intake ↓ fat accumulation ↓	[111]
Chronic Disease	Obesity	*UCP1*	Promoter	dCas9	VPR	(in vivo & in vitro) *UCP1* mRNA ↑		ND	(in vivo) body weight ↓ glucose tolerance ↑ insulin sensitivity ↑ weight rebound ↓	[112]
Chronic Disease	Type 2 diabetes mellitus	*Fabp4*	Promoter	dCas9	-	(in vivo & in vitro) *Fabp4* mRNA ↓		ND	(in vivo) body weight ↓ insulin sensitivity ↑ hepatic steatosis reversal	[113]
Chronic Disease	Hyper-cholesterolemia	*PCSK9*	Promoter	dCas9	KRAB, DNMT3A-DNMT3L	(in vivo & in vitro) *PCSK9* mRNA/protein ↓		ND	(in vivo) low-density lipoprotein cholesterol ~ 70% ↓ durability > 1 year (mice and non-human primates)	[114]
Chronic Disease	Hyper-cholesterolemia	*PCSK9*	Promoter	ZFP	KRAB, DNMT3A-DNMT3L	(in vivo) *PCSK9* secretion/protein ↓		ND	(in vivo) low-density lipoprotein cholesterol ~ 29% ↓ total cholesterol ~ 26% ↓ durability ~ 330 days	[115]
Chronic Disease	Hyper-cholesterolemia	*PCSK9*	Promoter	TALE	DNMT3A-DNMT3L	ND		ND	(in vivo) low-density lipoprotein cholesterol ↓ long-term efficacy maintained for ~ 343 days	[116]
Chronic Disease	Hyper-cholesterolemia	*PCSK9*	Promoter	dCas12i3	DNMT3A-DNMT3L	ND		ND	(in vivo) low-density lipoprotein cholesterol ~ 51.4% ↓ long-term efficacy maintained for ~ 180 days	[117]
Chronic Disease	Renal fibrosis	*RASAL1*,* Klotho*	Promoter	dCas9	TET3CD	(in vivo & in vitro) *RASAL1*/*Klotho* mRNA ↑		ND	(in vivo) kidney fibrosis attenuation	[118]
Lung disease	Pulmonary fibrosis	*Cxcl11*	Promoter	ZFP, dCas9	TET1CD, Thymine-DNA Glycosylase	(in vitro) *Cxcl11* mRNA ↑		(in vivo) airway interferon-γ response ↑	ND	[119]
Inflammatory	Acute Kidney Injury	*Klotho*	Promoter	Wild type-Cas9	p65, HSF1	(in vivo) *Klotho* mRNA ↑		(in vivo) inflammation ↓	(in vivo) renal function ↑	[120]
Inflammatory	Acute lung Injury	*CXCL1–8*	Promoter, Enhancer	TALE, ZFP	KRAB, MQ1	ND		(in vivo) airway inflammation ↓ immune cell infiltration ↓	(in vitro) neutrophil migration ↓ (in vivo) asthma phenotype improvement	[121]
Inflammatory	Inflammation-related diseases	*IL1RN*	Promoter	dCas9	DNMT3A	(in vitro) *IL1RN* mRNA ↓		(in vitro) myeloid reprogramming ↑ inflammatory response ↓	ND	[122]

a) AKT: protein kinase B

b) mTOR: mechanistic target of rapamycin

c) MAPK: mitogen-activated protein kinase

d) SAM: synergistic activation mediator

e) VEGF: vascular endothelial growth factor

f) PI3K: phosphoinositide 3-kinase

g) CAR-T: chimeric antigen receptor T cells

h) VMAT2: vesicular monoamine transporter 2

### Monogenic disorders

Epigenome editing can be used to treat monogenic disorders caused by haploinsufficiency or insufficient gene expression [123]. In cystic fibrosis (CF), mutant CFTR proteins are largely degraded before reaching the plasma membrane, resulting in reduced chloride ion channel activity [66]. Targeting the *CFTR* promoter with dCas9-VPR was able to compensate for reduced CFTR protein levels in patient-derived nasal epithelial cells homozygous for the deletion of phenylalanine at position 508 of CFTR. Partial restoration of reduced CFTR function was observed, especially when combined with FDA-approved CF therapies, such as VX-809 (lumacaftor) and VX-770 (ivacaftor) [66]. In Beckwith–Wiedemann syndrome, hypermethylation of imprinting control region 1 (ICR1) on the maternal allele causes silencing of *H19* and biallelic activation of *IGF2*. In wild-type HEK293 cells, targeting dCas9-TET1CD to the CCCTC-binding factor (CTCF) binding sites within ICR1 reduced DNA methylation up to a 90%, which led to increased CTCF occupancy. These epigenetic changes induced an approximately 1.8-fold increase in *H19* expression that was stably maintained for 27 days [67]. A similar case has been observed in Marfan syndrome, in which heterozygous mutations in the *FBN1* gene cause haploinsufficiency. When primary porcine fibroblasts derived from a Marfan syndrome model were treated with dCas9-TET1CD, demethylation was induced at the CpG island shore of the *FBN1* promoter, resulting in approximately 20% upregulation of *FBN1* mRNA compared to non-sgRNA controls [68].

Gene dysfunction in monogenic disorders can also be mitigated by activating the expression of alternative genes whose expression functionally compensates for causal gene dysfunction. In retinitis pigmentosa, rod cell degeneration caused by mutations in the *rhodopsin* gene can be partially compensated by *OPN1MW* expression [124, 125]. Activation of *Opn1mw* expression with dCas9-VPR delayed rod cell degeneration and successfully improved visual responses in rhodopsin-deficient (*Rho*+/−) retinitis pigmentosa mice [69].

Another example is Hutchinson–Gilford progeria syndrome (HGPS), in which mutations in *LMNA* gene lead to accumulation of progerin proteins and premature cellular aging. dCas9-SAM-mediated activation of *Oct4* ameliorated multiple aging-associated phenotypes in HGPS mice, including reduced progerin levels and age-marker expression, as well as improved tissue integrity and overall health [70, 126–129].

### Cancer

Epigenome editing systems have been used to restore the expression of tumor suppressor genes, including *PTEN*, *REPRIMO*, *SERPINB5*, *ZAR1*, *BRCA1*, *BARD1*, *miR-524*, *miR-200c*, *SARI*, and *EPHA* [71–76, 80, 82, 83]. Recent studies in cancer have explored multiplexed epigenome editing approaches since cancer involves dysregulation of multiple genes. In Hep3B cells, simultaneous activation of *HHIP*, *MT1M*, *PZP*, and *TTC36* using dCas9-SAM and four sgRNAs suppressed cancer proliferation and migration [78]. Although many studies have demonstrated the feasibility of regulating gene expression in vitro, a subset has extended these findings to in vivo models and successfully shown amelioration of cancer phenotypes. One example is the in vivo delivery of dCas9-VP64 in pH-sensitive multistage delivery nanoparticles, which selectively activated *miR-524* expression in tumor tissues and reduced tumor weight by up to 60% in an MDA-MB-231 xenograft mouse model [75].

The epigenome editor OTX-2002, designed to downregulate *MYC* expression using two ZFP fused to the bacterial DNA methyltransferase MQ1 and KRAB, respectively, entered the clinical trial (NCT05497453) in patients with hepatocellular carcinoma and other solid tumor known for association with the *MYC* oncogene in 2022. It is the first human application of epigenome editing-based therapeutics [79]. While the company terminated the OTX-2002 Phase1/2 clinical trial and temporarily put its clinical development on hold, the OTX-2002 case showed potential as a viable therapeutic option for cancer treatment.

Epigenome editing has also expanded its application to the development of cancer immunotherapy, particularly to improve chimeric antigen receptor (CAR) T cells persistence by suppressing genes associated with T cell exhaustion [130–133]. For example, KRAB-TALE-DNMT3A-Dnmt3L-mediated silencing of *PDCD1* and *LAG3* maintained CAR T cell effector functions during prolonged stimulation [134, 135]. Similarly, DNMT3A-DNMT3L-dCas9-KRAB-mediated repression of RASA2 enhanced antigen sensitivity, tumor killing capacity, tumor control, and survival in B-cell malignancy models [84].

### Neurological diseases

#### Neurodegenerative disorders

In Alzheimer’s disease (AD) models, epigenome editing techniques have been used to modulate the expression of pathogenic hippocampal genes associated with synaptic dysfunction, including *Dlg4*,* Per1*, *Syt1*, *ApoE*, and *Gad1* [87–90]. Several studies have reported improvements in memory and cognitive performance following targeted epigenetic regulation in mouse models [86, 88]. Stereotaxic delivery of ZFP-VP64 targeting the *Dlg4* promoter into transgenic AD mice resulted in sustained behavioral improvements in memory that persisted for up to five months [86]. Similarly, selective repression of *Syt1* expression in the dentate gyrus of adult mice using dCas9-KRAB associated with behavioral evidence of cognitive improvement [88].

Epigenome editing has successfully reduced the accumulation of toxic protein aggregates in various models of neurodegenerative diseases, including Parkinson’s disease (PD), Huntington’s disease (HD), and prion diseases. *SNCA* is the gene encoding α-synuclein, a presynaptic protein that aggregates abnormally and impairs synaptic vesicle dynamics in familial and sporadic PD [136]. In iPSC-derived neurons from patients carrying *SNCA* triplication, dCas9-KRAB targeting the *SNCA* promoter reduced total α-synuclein protein levels by up to 60%, and this was accompanied by a marked alleviation of mitochondrial DNA damage and oxidative stress [94]. HD is caused by expanded CAG repeats in exon 1 of *HTT*, leading to the production of mutant HTT (mHTT). mHTT forms toxic aggregates within cells, particularly in the nucleus and perinuclear regions, and disrupts transcription, axonal transport, and mitochondrial function [96]. When ZFP-KRAB, which recognizes the expanded CAG repeat region, was delivered into patient-derived neuronal cells, mHTT protein levels were reduced by approximately 50% compared to those in untreated controls, whereas wild-type HTT protein expression remained unchanged. Extending this system to an HD mouse model restored striatal neuronal signaling and improved motor performance. In prion diseases, *Prnp* encodes PrP, a misfolded protein that propagates through templated conformational conversion and induces neurotoxicity in the brain [137]. To suppress *Prnp* expression, a ZFP platform fused to DNMT3L, and the histone H3 tail domain (ZFP-DNMT3L-H3 tail) were designed to target the *Prnp* promoter. AAV-mediated delivery of this system to adult mice reduced *Prnp* mRNA levels by up to 90% and PrP levels by 80% throughout the brain. To mitigate the risks associated with prolonged AAV-mediated transgene expression, a self-silencing epigenome editing system was developed that autorepresses its own expression after target gene silencing. This strategy maintained *Prnp* repression for at least 13 weeks without detectable toxicity [97].

#### Neurodevelopmental disorders

Epigenome editing has been actively applied to monogenic neurodevelopmental disorders caused by or associated with abnormal gene inactivation, including *FMR1* silencing in Fragile X syndrome, *SCN1A* haploinsufficiency in Dravet syndrome, *C11orf46* haploinsufficiency in callosal hypoplasia, *MECP2* inactivation in Rett syndrome, maternal *UBE3A* loss in Angelman syndrome, and paternal *SNRPN* deficiency in Prader-Willi syndrome [98–101, 104, 138]. In the study of Rett syndrome (RTT), a multiplex epigenome editing was performed in which DNA demethylation and insulation loop formation were combined at a single *MECP2* locus to restore *MECP2* expression and insulate the edited *MECP2* locus from the repressive chromatin environment that could limit DNA demethylation-mediated activation. Specifically, the *MECP2* promoter was demethylated using dCas9-TET1CD, and a chromatin loop was formed by CTCF-fused dCas12a recruited to two CTCF anchor sites flanking the *MECP2* locus. This engineered insulation loop increased MECP2 protein expression by approximately 20% compared to dCas9-TET1CD alone in RTT neurons, doubled the neuronal firing rate, and maintained these effects for up to 57 days after differentiation. Moreover, electrophysiological parameters were fully restored to wild-type levels [100].

#### Neuromuscular disorders

Duchenne muscular dystrophy (DMD) is a progressive muscle wasting disorder characterized by the absence of dystrophin, encoded by the *DMD* gene. *DMD* is the largest single gene in the human genome spanning approximately 2.4 Mb. Due to the wide mutational spectrum in the *DMD* gene, approaches that directly correct or target individual mutations have practical limitations [139]. In 2023, the AAV-based gene therapy, delandistrogene moxeparvovec (ELEVIDYS), received accelerated FDA approval for the treatment of DMD. This therapy circumvents the packaging capacity limitation of AAV vectors by delivering a truncated form of dystrophin, called microdystrophin, which contains only the essential functional domains [140, 141]. However, its function is not as complete as the full-length dystrophin [141]. A promising alternative is the activation of utrophin, a functional homolog encoded by *UTRN* that can ameliorate the pathological features of DMD [142–144]. In DMD mouse models, delivery of a dCasMINI-VPR-sgRNA system targeting the *Utrn* promoter restored muscle strength, cardiac function, and motor performance to levels similar to those of wild-type mice, with therapeutic effects persisting for at least six months [109]. This system incorporates several features to enhance in vivo delivery and tissue specificity. dCasMINI is a miniaturized Cas-derived DBD that is compatible with all-in-one AAV packaging. Delivery was achieved using MyoAAV, an engineered vector with enhanced muscle tropism that achieves higher transduction efficiency in both skeletal and cardiac muscles than conventional AAV9 [145]. Furthermore, the use of muscle-specific promoters restricts dCasMINI VPR expression to muscle tissues, further improving tissue specificity. When the same system was applied to healthy cynomolgus monkeys, utrophin protein levels increased in the sarcolemma, without evidence of immune-related adverse effects [109].

The applicability of epigenome editors in neuromuscular disorders has also been demonstrated in congenital muscular dystrophy type 1A (MDC1A). MDC1A characterized by compromised muscle stability and peripheral nerve myelination is caused by non-functional laminin-α2. The functional deficiency of laminin-α2 can be compensated by structurally similar laminin-α1 encoded by *LAMA1* [146]. In the MDC1A mouse model, VP64-dCas9-VP64 led to a more than 9-fold increase in *Lama1* expression in skeletal muscle and the sciatic nerve compared to untreated controls, along with an approximately 50% reduction in muscle fibrosis and an increase in specific tetanic force. In addition, nerve conduction velocity and hind limb mobility improved, and by the end of treatment, hind limb paralysis had resolved with marked recovery of motor function. Notably, although MDC1A has long been regarded as an intractable disease with irreversible muscle dysfunction, the therapeutic benefit of VP64-dCas9-VP64 has been observed even in symptomatic three-week-old mice [107].

### Chronic diseases

Epigenome editing may provide a durable approach for obesity by reducing the need for repeated dosing. In obesity models, AAV-mediated delivery of dCas9-VP64 to the hypothalamus increased expression of appetite-regulating genes, including *Sim1* and *Mc4r*, leading to sustained body-weight reduction for more than nine months and eight weeks, respectively [111]. In addition, adipocyte-targeted delivery of a CRISPRi system using an adipocyte-targeting sequence (ATS) reduced *Fabp4* expression, suppressed lipid accumulation, and decreased body weight by approximately 20% while improving insulin sensitivity in diet-induced obese mice [113].

Epigenome editing also offers a potential alternative to current anti-PCSK9 therapeutics for the treatment of hypercholesterolemia since a single administration could achieve long-term maintenance of low-density lipoprotein cholesterol (LDL-C) levels [147, 148]. Specifically, lipid nanoparticle (LNP) delivery of ZFP–DNMT3A–DNMT3L–KRAB targeting the *Pcsk9* promoter in adult mice resulted in durable *Pcsk9* repression lasting for more than 330 days and a 35% reduction in LDL-C levels [114]. Remarkably, *Pcsk9* silencing was maintained even after a partial hepatectomy and subsequent liver regeneration [114]. In a similar study, LNP-mediated delivery of dCas9–DNMT3A–DNMT3L–KRAB targeting the *PCSK9* promoter into human *PCSK9* transgenic mice resulted in sustained *PCSK9* silencing for over one year and a concomitant reduction of plasma cholesterol. These results were further validated in *Cynomolgus macaques*, where it dose-dependently repressed *PCSK9* expression and lowered LDL-C levels by up to 70% without observable adverse effects, with the benefits persisting for at least three months [115]. TALE-based epigenome editors likewise achieved > 90% *PCSK9* repression and durable LDL-C reduction in nonhuman primates following a single administration [116].Collectively, the consistent and durable silencing of *PCSK9* across ZFP-, dCas9-, and TALE-based platforms highlights its potential as a leading clinical target for epigenome editing therapeutics.

## Challenges and future of epigenome editing technology

Epigenome editing systems have emerged as powerful gene regulation tools with promising therapeutic potential for a wide range of diseases ranging from monogenic disorders to complex chronic diseases. However, most epigenome editing applications remain at the proof-of-concept stage. Key challenges include achieving tissue-specific delivery, ensuring durable and safe long-term effects, and evaluating immunogenicity. Additionally, the reversibility and persistence of epigenetic modifications, particularly in heterogeneous tumors and post-mitotic neuronal tissues, requires further investigation to define the long-term consequences and safety of epigenome editing. In this section, the key obstacles and future directions for advancing epigenome editing toward clinical applications are discussed.

### Toward precise and safe epigenome editing: durability, reversibility, efficiency, and specificity considerations for clinical application

A major challenge of epigenome editing is the variability in its editing durability across target loci and cell types since gene expression is regulated by distinct chromatin environments in different cellular contexts [65, 149, 150]. For example, the KAL system, consisting of KRAB-dCas9, DNMT3A-dCas9, and DNMT3L overexpression, exhibited marked differences in editing durability between a colorectal cancer cell line and a prostate cancer cell line [150]. In HCT116 cells, KAL induced transient *HER2* repression, with expression returning to baseline by day 14, whereas sustained *HER2* repression was maintained in LNCaP [150]. Subsequent studies revealed that these differences in editing durability are affected by the pre-existing chromatin landscape at the target locus, including promoter H3K27ac levels and the coexistence of H3K4me3 and H3K27me3 [151, 152].

Editing efficiency can also be affected by the local TF motif context. A systematic assessment of how identical effectors function in different TF motif contexts showed that gene repression mediated by the SunTag system using 5xRing1b-scFv or 5xG9a-scFv was less efficient in the absence of YY1 motifs, whereas repression by SunTag-5xSetd2-scFv was reduced in the absence of CTCF motifs [152]. These observations demonstrate that local TF motifs can either buffer or potentiate the activity of epigenome editors.

The balance between durability and reversibility represents another important consideration when selecting an effector module. DNA methylation is generally more stable than other epigenetic modifications. This stability may provide long-term therapeutic benefit, making DNA methylation-based editing attractive for chronic diseases. However, persistent transcriptional changes may become problematic if the regulatory outcome is excessive, associated with unpredicted long-term effects, or occurs in non-target cells and tissues. Therefore, the duration and extent of epigenetic changes induced by different epigenome editors, target loci, and cell types should be carefully characterized.

Specificity remains a critical challenge. Although considerable efforts have focused on minimizing off-target effects, unintended chromatin modifications beyond the target loci could potentially affect neighboring genes. Since chromatin modifications contribute to higher-order genome organization, epigenome editing may also influence long-range enhancer-promoter interactions, resulting in broader transcriptional consequences than anticipated. Comprehensive genome-wide analyses of chromatin accessibility, histone modifications, DNA methylation, three-dimensional chromatin interactions, and transcriptional responses will therefore be essential for evaluating the specificity of therapeutic epigenome editors.

### Delivery systems for in vivo epigenome editing

An epigenome editing construct is typically 6–7 kb in size, which exceeds the packaging capacity of AAV vectors [153]. Early in vivo delivery therefore relied on dual-AAV systems in which the editor is divided between two vectors and reconstituted in vivo [69, 154–156]. However, these systems are inherently constrained by the requirement that both vectors should be delivered to the same cell, an event that occurs with relatively low efficiency in vivo. Increasing vector doses to improve co-transduction is challenging because high AAV titers can trigger unwanted immune responses against both the viral vector and the expressed dCas proteins [157, 158]. Recent studies on all-in-one systems have focused on minimizing the size of epigenome editing cassettes so that they can be packaged into a single AAV vector [89, 109]. More compact dCas proteins, such as *Staphylococcus aureus*-derived dCas9 (dSaCas9) and the engineered Cas12f variant dCasMINI, have been developed [89, 109]. These smaller dCas molecules show comparable activity to that of conventional dCas9-based systems [159, 160].

Another barrier for in vivo delivery of epigenome editing tools is tissue-specific delivery. Various AAV capsids with defined tissue tropism have been developed, including a muscle-tropic AAV9 variant, MyoAAV, which exhibits more than 10-fold or 6-fold higher delivery to skeletal and cardiac muscle, respectively, compared with non-muscular tissues [69, 99, 109, 145, 161]. Additionally, the use of tissue-specific promoters to drive expression of epigenome editors can further enhance tissue specificity [88, 109, 140, 141, 162].

Non-viral vectors have emerged as a promising strategy for in vivo delivery of epigenome editing systems since they can accommodate large cargos while exhibiting lower immunogenicity and improved safety profiles. LNPs are the most widely used and can be engineered for tissue-specific delivery through lipid modification or incorporation of targeting ligands [79, 114–116, 163]. OTX-2002, the first epigenome editing therapy that entered clinical trials, is delivered via LNPs [79].

Neutral 1,2-dioleoyl-sn-glycero-3-phosphocholine (DOPC)-based nanoliposomes also shown efficient nucleic acid delivery and favorable safety profiles in preclinical studies [164, 165]. A siRNA-based therapy utilizing DOPC nanoliposomes has advanced to a Phase I clinical trial (NCT01591356) [166]. As a vector-free approach, recombinant epigenome editor proteins can be directly administered via intranasal instillation or nebulized aerosol inhalation [119].

### Multi-gene targeting epigenome editing

**Fig. 4:**
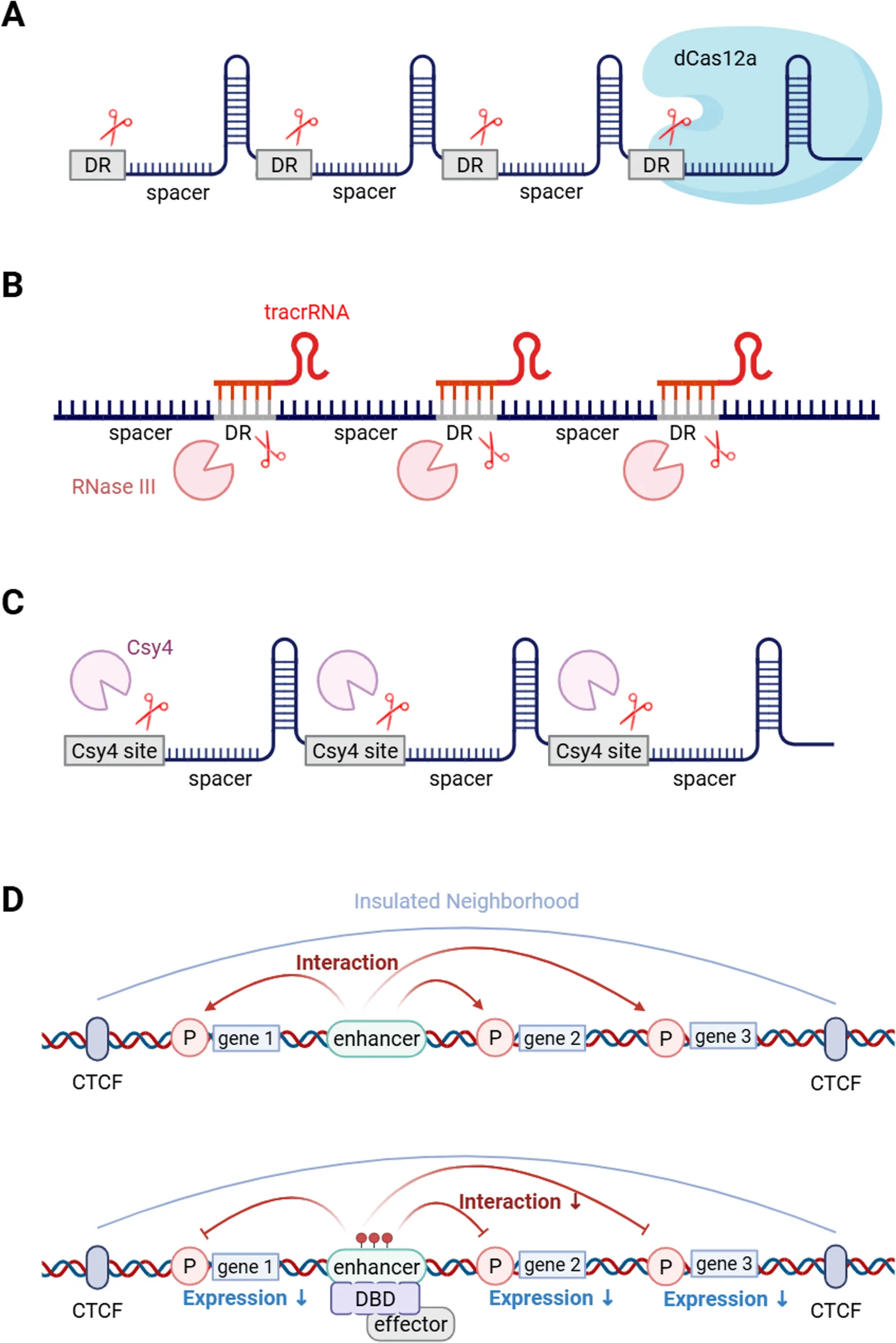
Diverse strategies of multi-target epigenome editing. (**A**) Cas12a-based self-processing system uses direct repeats (DR) to generate individual crRNAs. (**B**) RNase III-based system requires tracrRNA to pair with DR in pre-crRNA, forming double strand structure for RNase III cleavage. (**C**) Csy4-based system uses Csy4 recognition sites inserted between gRNAs to allow for Csy4 to cleave and release individual gRNAs. (**D**) Epigenome editing of enhancer can simultaneously regulate multiple genes (P: promoter, DBD: DNA-binding domain)

Multitarget epigenome editing tools capable of simultaneously modulating multiple genes or regulatory elements are likely to be useful in addressing complex diseases, including neurological disorders, cancers, and metabolic diseases [114, 115]. The most intuitive approach is the delivery of multiple sgRNAs. However, this strategy is limited by inefficient co-delivery and variable sgRNA expression levels [167]. To overcome these limitations, gRNA array–based systems have been developed, in which multiple sgRNAs are transcribed as a single polycistronic transcript and subsequently processed into individual gRNAs within the cell [168–171]. A representative example is a dCas12a-based system that leverages the intrinsic RNase activity of dCas12a to cleave a long CRISPR RNA (crRNA) array into individual crRNAs (Fig. 4A) [168–170, 172]. A dCas9-based system co-expressing trans-activating CRISPR RNA (tracrRNA) and RNase III along with a gRNA array has been shown to mimic the gRNA processing process of dCas12a-based system (Fig. 4B) [171, 173, 174]. A similar approach involves the use of the exogenous endoribonuclease Csy4 derived from the Type I-F CRISPR system that recognizes specific stem-loop structures formed by repeat sequences in a CRISPR array and cleaves a polycistronic gRNA array transcript into individual functional gRNAs (Fig. 4C) [174].

Despite these advances, achieving robust regulation of multiple targets remains challenging when effector proteins do not stably occupy each target locus. To address this limitation, liquid–liquid phase separation has been incorporated into dCas12a-based editors through fusion with intrinsically disordered regions (IDRs), creating locally concentrated condensates at target sites. For example, dCas12a-IDR-VP64 resulted in more than 10-fold increase in gene activation compared to dCas12a-SunTag-VP64 [175, 176].

Another approach is to modulate key enhancers that regulate the expression of gene clusters or multiple genes within insulated neighborhoods **(**Fig. 4D**)**. When ZFP-KRAB was targeted to the key regulatory region of *CXCL1–8* gene clusters, enhancer–promoter looping was disrupted, leading to the suppression of promoter activity of multiple chemokine genes, including *CXCL1*, *CXCL2*, *CXCL3*, and *CXCL8* [121].

## Conclusions

Epigenome editing represents not only a powerful research tool for investigating the effects of epigenetic modifications and identifying functional gene regulatory elements but also a promising therapeutic modality with the potential to overcome key limitations of current gene therapies, including off-target effects and irreversible DNA alterations. Recently, the translation progress of this technology has advanced into clinical trials. In addition to OTX-2002, the first-in-class chronic hepatitis B silencer TUNE-401, which employs LNP-delivered epigenome editing-mediated silencing of both integrated and episomal hepatitis B virus genomes, has entered a Phase 1b clinical trial (NCT06671093). Similarly, CRMA-1001, which employs a comparable epigenetic silencing strategy for chronic hepatitis B, is being evaluated in a Phase 1/2 clinical trial (NCT07200193). EPI-321, an AAV-delivered epigenetic editing therapy that represses the pathogenic *DUX4* gene, is also currently being evaluated in a Phase 1/2 trial for facioscapulohumeral muscular dystrophy (NCT06907875).

Primary endpoints in these early clinical studies mainly focus on safety and tolerability. Additional endpoints, including target site epigenetic modifications, changes in target gene expression and downstream markers, off-target effects, and immunogenicity, will also be important for evaluating the efficacy and safety of epigenome editing therapeutics. Continued progress toward clinical translation will require a deeper understanding of epigenetic regulatory mechanisms and disease-specific chromatin dynamics, as well as innovations in efficient and targeted in vivo delivery technologies.

## Data Availability

No datasets were generated for this manuscript.
